# Oral and Head and Neck Cancers in Israel in the Paediatric Population, 1970–2017: A Retrospective Epidemiological Study

**DOI:** 10.3390/children13020269

**Published:** 2026-02-14

**Authors:** Rachail Meiseles, Lital Zecharyahu, Avraham Zini, Esti Davidovich

**Affiliations:** 1 Department of Paediatric Dentistry, Hadassah School of Dental Medicine, Hebrew University of Jerusalem, Jerusalem 91120, Israel; 2 Institute of Biomedical and Oral Research, Faculty of Dental Medicine, Hebrew University of Jerusalem, Jerusalem 91120, Israel; 3 Department of Community Dentistry, Hadassah School of Dental Medicine, Hebrew University of Jerusalem, Jerusalem 91120, Israel

**Keywords:** head and neck neoplasm, paediatric oral cavity malignancies, pedodontics, epidemiologic study

## Abstract

Background: Oral malignancies in the paediatric population are rare, but if left untreated, the patient’s prognosis may be altered. The current literature is varied in its findings regarding common sites and types of tumours found in the paediatric population. Our goal was to describe the distribution of paediatric head and neck cancers, test associations, quantify temporal trends and perform survival analyses. We then compared our results with the current literature. Methods: Approval for the study was given by the Hadassah Medical Organization Helsinki Committee (HMO0792-20). We obtained data from the Israel National Cancer Registry, for the years 1970–2017, regarding head and neck malignancies, including oral malignancies, diagnosed in Israel in individuals under the age of 19. We performed a comprehensive statistical analysis, including annual incidence analysis, Kaplan–Meier survival curves to estimate 5-year survival, and a multivariable Cox proportional hazards model to evaluate the association between demographic and tumor-related variables and overall survival. Results: Our study consisted of 393 cases. The most common cancer location was the nasopharynx, and the most common cancer histology was of epithelial origin. Regarding gender, there were significantly more males diagnosed with cancer. Over 40% of the diagnosed cases were in the 15–18 age group. There was no evidence of a notable change in the average incidence rate over time. The lowest survival rates were observed in cancers originating in the oropharynx and in hematologic malignancies when stratified by tumor location and histology, respectively. Conclusions: The importance of this research is significant, as it adds to the current pool of information and touches on aspects that have not been commonly analysed.

## 1. Introduction

Worldwide oral cancer is the sixth most common malignancy. The main etiological factors include tobacco consumption and alcohol abuse. In addition, human papillomavirus (HPV), and HPV16 in particular, have been known to increase the risk of developing squamous cell carcinoma (SCC), especially in younger patients [1]. Further etiological factors that have been suggested to include environmental factors such as heavy smoking, congenital abnormalities and genetic dysregulation [2]. The majority of oral cancer cases occur in the fifth to eighth decades [1].

Under 10% of oral tumours are malignant and are rare in the paediatric population. According to the National Institution of Health (NIH), in 2008, the age-adjusted incidence for patients under 20 years old was 0.2 per 100,000. However, these numbers are rising, which is consistent with the increase in HPV infections [3].

The current literature is varied in its findings regarding the common sites and types of tumours found in the paediatric population, and the ages in which they manifest.

Cesmebasi et al. (2014) found that in patients aged 0–19, the common locations (in descending order) were 1—salivary glands, 2—nasopharyngeal, 3—nose, nasal cavity and middle ear, 4—gum and other mouth tumours, and 5—tongue [2]. Grønhøj et al. (2018) showed that in a Danish population aged 0–15, the common sites were the thyroid and soft tissue, followed by the oral cavity, pharynx and salivary glands [4]. According to Dhanuthai et al. (2018), among children aged 0–16, the most common sites (in descending order) were mandible, palate, gingiva, alveolar mucosa, maxilla and tongue [1]. In contrast, the findings of Vaibhav et al. (2018) showed that the common sites in patients aged 2–15 (in descending order of frequency) were tongue, buccal mucosa, lip and palate [5].

Sengupta et al. (2009) found that the most common symptom of head and neck cancers was the presence of a painless enlarged neck mass. Other symptoms included nasal masses, epistaxis, and orbitofacial swelling [6].

Arboleda et al. (2020) analysed the commonly diagnosed paediatric head and neck histological subtypes by geographic location in their review. They found that in Europe, the common diagnosis was lymphoma, and sarcoma, specifically rhabdosarcoma, had a substantial part in the increase in incidence. In Asia, carcinoma was the main histological diagnosis, while in South America and Africa lymphoma was the most common, followed by carcinomas and sarcomas. In North America, carcinomas were the most prevalent, followed by lymphomas and sarcomas [7]. Grønhøj et al. (2018)’s study found that the common histology diagnosis was rhabdomyosarcoma, followed by papillary thyroid carcinomas and mucoepidermoid carcinomas [4].

Dhanuthai et al. (2018) states that SCC is the most common tumour in the paediatric population, followed by mucoepidermoid carcinoma and lymphoma [1]. Yuhan et al. (2018) lists several malignancies found in paediatric patients, including Lymphoma, both Hodgkins and non-Hodgkins, whose incidence is highest amongst children younger than 5 years and teenagers. More examples include rhabdomyosarcoma, a soft tissue tumour, and osteosarcoma, a bone tumour, which on average present at 15 years old. Other malignancies mentioned are SCC, which is described as rare, histiocytosis, and malignancies of minor salivary glands [8]. Morse et al. (2018) describe the tumours present in salivary glands in those aged 19 years and younger and their study found that the most common tumours (in descending order) were in the parotid, submandibular, sublingual glands [9].

Person L et al. (2021) conducted a study of paediatric head and neck cancers (of patients aged 0–15) based on the cancer registry in France, and found the survival over a 5-year period of time was over 85% for lymphomas, neuroblastomas, germ cells tumours, carcinomas and melanomas. The 5-year survival rate was 65–75% for osteosarcomas and rhabdomyosarcomas, and 29% for malignant rhabdoid tumours [10].

In this paper, we researched the data from the Israel National Cancer Registry, 1970–2017. We described the distribution of paediatric head and neck malignancies (ICD-O C00-C14) [11] in Israel by site and histology, tested associations between site/histology and sex and age groups (and descriptive comparisons by ethnicity and country of birth), quantified temporal trends using incidence-based models and described survival differences by site and histology and associated variables with hazard ratio. We then compared our results with the current published research.

## 2. Materials and Methods

Our study is a retrospective epidemiological study. The data used in this study was obtained via the Israel National Cancer Registry, which lists all cancers diagnosed and treated in Israel since 1970. Our data included all head and neck cancers of paediatric patients (aged 0–18) diagnosed and treated in Israel between the years 1970 and 2017 and consisted of 393 cases. We received the required approval to utilise the human subject data in this study from the Hadassah Medical Organization Helsinki Committee (HMO-0792-20).

The variables included in the data were diagnosis age, year of birth, year of death, death age, biological gender, nationality, country of birth, cancer location, histology of cancer and stage of cancer. The stage of cancer at the time of diagnosis was not examined in this paper due to missing data in over 60% of the cases.

Prior to analysis, the variables were classified into subgroups. Cancer sites were grouped according to The International Classification of Disease for Oncology (ICD-O) location codes for head and neck malignancies [11], described as codes C00-C14, along with their general behaviour patterns (malignant vs. benign): 1—Oral cavity, including lip, gums, floor of mouth, tongue, and hard palate. 2—Oropharynx, including tonsils, base of the tongue, and soft palate. 3—Parotid and other major salivary glands. 4—Nasopharynx. 5—Other. Histological origin was grouped in accordance with ICD-O histology codes for head and neck malignancies [12,13] (the data consists of codes within the range of 80001 and 99701): epithelial origin, gland-associated tumours, connective tissue, muscle, nerve-associated, hematologic malignancies, and other. Diagnosis age was grouped as: under 6, 6–10, 10–13, 13–15, 15–17 and over 17 years of age. Biological gender was classified as male and female. Nationality was defined as Jewish and other. Country of birth was defined as Israel and other.

Annual population counts for Israel from 1970 to 2017 were obtained from the “World Bank” database [14]. The number of newly diagnosed cases per calendar year was calculated directly from the study dataset based on the year of diagnosis. To account for population growth over time, population size was incorporated as an offset. Temporal trends in cancer incidence were evaluated using negative binomial model (NB), with the calendar year entered as a continuous predictor. Results are reported as incidence rate ratios (IRRs) with 95% confidence intervals (CIs) and corresponding *p*-values.

Kaplan–Meier curves were used to calculate survival for a 5-year period. The multivariable Cox proportional hazards model was used to evaluate the association between demographic and tumour-related variables and overall survival. Hazard ratios (HRs) with 95% confidence intervals (CIs) were reported. The proportional hazards (PHs) assumption was assessed using Schoenfeld residuals test.

Statistical significance was defined as *p* < 0.05, and 2-sided tests were performed. The statistical analysis was conducted using IBM SPSS Statistics 26 and using Python 3.12.

## 3. Results

Between the years 1970 and 2017, there were a total of 393 new cases diagnosed with head and neck cancer in the Israeli paediatric population (ages 0–18). Table 1 presents the distribution of the cancer location with respect to gender, age, ethnicity and origin. The most common cancer site was the nasopharynx (41%), followed by the major salivary glands (21%), oropharynx (15%), oral cavity (14%) and other sites (9%). The nasopharynx consistently held the majority of cases within all categories, except two, one being the 6–10 age group, and the other cases originating in eastern Europe. There were significantly (*p* < 0.001) more male cases than female cases, 60% vs. 40%, respectively. With regard to age, there was a statistically significant relationship (*p* < 0.001), with close to 40% of cases between 15 and 18 years old. Regarding nationality, 80% of the cases were Jewish. The most common country of origin was Israel, with 68% of the sample; 26% were of unknown origin, followed by America/western Europe/Australia (4%), Asia/Africa (1.3%) and eastern Europe (0.3%).

Table 2 describes the relationship of cancer histology with gender, age, ethnicity and origin. Cancers of epithelial origin were the most common (33%), followed by hematologic malignancies (25%), gland-associated tumours (22%), muscle and other (both 8%), connective tissue (2%) and nerve-associated (1%). There was a significant association between the histology type and gender (*p* < 0.001). The most common histology type amongst males was of epithelial origin, and amongst females was gland-associated tumours. Regarding age distribution, hematologic malignancies and muscle origin were the most common in the younger age groups (<10), while cancers of epithelial origin and gland-associated tumours were the most common in the older age groups (13–18). Amongst both Jews and other ethnicities, the most common cancer was of epithelial origin. As stated earlier, Israel was the largest country of origin of this sample, followed by an unknown country of origin. Within these groups, cancers of epithelial origin were again the most common.

**Figure 1:**
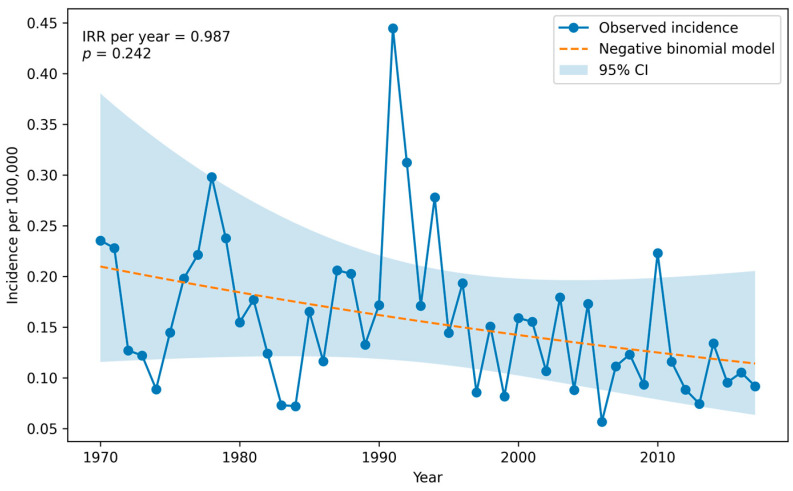
Annual incidence rate per 100,000 of total population.

Figure 1 illustrates the annual incidence rate of newly diagnosed paediatric head and neck cancers per 100,000 of the total population between 1970 and 2017, along with model-based estimates and their 95% confidence intervals. Calendar year was not significantly associated with incidence rates over the study period (IRR per year = 0.98, *p*-value = 0.24). Although a slight decrease in incidence was suggested, the effect size was small and not statistically significant, indicating no evidence of a notable change in the average incidence rate over time.

**Figure 2:**
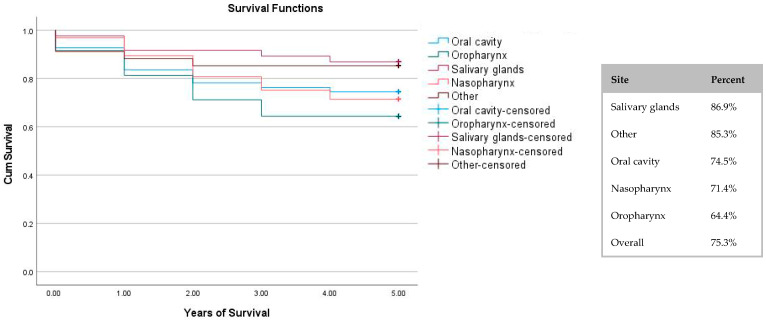
Five-year survival chart by cancer location. Censoring is represented by a + sign; it represents unknown death up to the time the data was filed.

Figure 2 presents the 5-year survival rate by cancer location. It demonstrates that the highest survival rate was reported for salivary gland tumours (86.9%), while the lowest survival rate was for oropharynx tumours (64.4%). The overall survival rate over the 5-year period was 75.3%.

**Figure 3:**
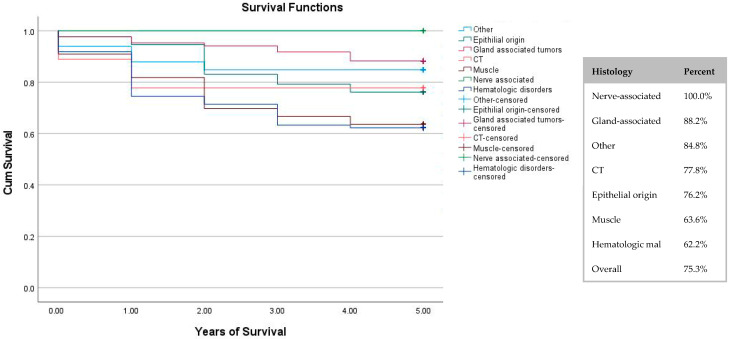
Five-year survival chart by histological group. Censoring is represented by a + sign; it represents unknown death up to the time the data was filed.

Figure 3 shows the 5-year survival group by histological group. Nerve-associated tumours had the highest survival rate (100%), though there were only five cases in the data. Hematologic malignancies had the lowest survival rate (62.2%).

Table 3 presents a multivariable Cox proportional hazards model table. A multivariable Cox model was fitted, including age at diagnosis, gender, tumour site, and histologic group. Age at diagnosis was not associated with overall survival (HR 1.00, *p*-value = 0.95). Similarly, no significant association was observed for gender (HR 1.05, *p*-value = 0.86). Tumour site was not associated with survival, and none of the site subgroups showed a statistically significant difference compared with the reference category (oral cavity). Regarding histology, two histologic subgroups (muscle and hematologic malignancies) demonstrated an increased hazard of death compared with the reference group (other) (HR 5.56, *p*-value = 0.038; HR 4.04, *p*-value = 0.07). The remaining histologic groups showed either increased or decreased hazard estimates; however, none of these histology associations reached statistical significance.

## 4. Discussion

This study consisted of 393 cases of malignancies that were characterised as ICD-O C00-C14 (head and neck, including oral malignancies), between 1970 and 2017, among children aged 0–18 in Israel. To provide context, the Israeli paediatric population comprised 1,183,300 children in 1970, and 2,851,900 children in 2016 [15].

For ethical reasons and to ensure confidentiality, the data was anonymized, and access was restricted to authorised researchers only.

The distribution of tumour location, from most to least common, was as follows: nasopharynx (41%), followed by the major salivary glands (21%), oropharynx (15%), oral cavity (14%) and other sites (9%). Similarly, Arboleda et al. (2022), who analysed data from a Brazilian centre and included 253 patients in their study, found that the nasopharynx was the main anatomical location for head and neck cancers (28.9%) [16]. This distribution is somewhat supported by Cesmebasi et al. (2014), who analysed the data from a US registry, which included 17 registered areas. Their study consisted of 1088 head and neck cancers among the paediatric population aged 0–19. They found that, in descending order, the most common location was the salivary gland, followed by nasopharyngeal tumours, nose, nasal cavity and middle ear, gums and other mouth tumours, and tongue [2]. In contrast, Grønhøj et al. (2018), who analysed data from the Danish cancer registry of patients aged 0–14 that included 169 cases, showed that the most common cancer location was the thyroid (although the thyroid was not included in our data), followed by soft tissue, oral cavity, pharynx and salivary glands [4].

The distribution of tumour histology, from most to least common, was as follows: cancers of epithelial origin (33%), hematologic malignancies (25%), gland-associated tumours (22%), muscle and other (both 8%), connective tissue (2%) and nerve-associated (1%). These findings are supported by Arboleda et al. (2020), in that carcinomas (epithelial origin) and lymphomas (hematologic malignancies) are amongst the most commonly diagnosed head and neck cancers in the paediatric population. Sarcomas (connective tissue origin), on the other hand, were not as common in Israel in the studied population as they appear to be in other places worldwide [7].

With regard to gender, there were 60% male cases versus 40% female cases. This was consistent with Daniel et al.’s (2025) umbrella review, which showed a higher prevalence amongst males [17].

Regarding age, 39% of cases were between ages 15 and 18. Younger age groups consisted mostly of hematologic cancers, while in the older age groups the most common diagnosis was of epithelial origin. The most common location in most of the age groups was the nasopharynx. Grønhøj et al. (2018) found that the most common cancer in the 0–9 age group was sarcomas, while thyroid carcinomas were the most common in the 10–14 age group [4]. Arboleda et al. (2020) state that it is possible that patients between the ages 10 and 19 may be more likely to be affected by head and neck cancers [7], our findings support this assumption.

Regarding ethnicity and country of origin, we had no statistically significant results. Cesmebasi et al. (2014) found that paediatric head and neck tumours were more common amongst the white ethnic population, while nasopharyngeal tumours were more common amongst the black population [2], yet it is difficult to compare these results to ours as this was not data that we were privy to in our registry.

We found no statistically significant change in the average incidence rate over time. This finding is supported by Clements K et al. (2025), as they found a stable incidence of head and neck cancers over 20 years, though there was an overall increase in oropharyngeal cancers and a decrease in laryngeal cancers [18]. These findings stand in contrast to Board PP. (2022), who found that the incidence of oral cavity and pharynx cancers increased in adolescent and young adult females, although our incidence evaluation was not directed solely to oral cancers, and perhaps this can explain the difference [3]. Further research is recommended in order to evaluate trends.

Survival rates for a period of 5 years were calculated both by cancer site and by cancer histology. When evaluating the location of the cancer, we found that salivary gland tumours had the highest survival rates, while oropharynx tumours had the lowest survival rates. Modh et al. (2018) found that the overall survival rate over 5 years was 74%, which was similar to the overall survival in our study, which was 75.3%, though their distribution by subsite differed from ours [19]. Guan et al. (2024) found that nasopharyngeal cancers were associated with significantly better survival compared with oral cavity cancers [20], while our study showed a similar survival of 71.4% and 74.5%, respectively. Both of those studies included solely SCCs. According to Daniel JB et al. (2025)’s umbrella review, overall survival of head and neck malignancies in sites other than the oral cavity were higher [17].

Evaluation of the cancer histology showed that nerve-associated tumours had a 100% survival rate, though this group consisted of only five cases and we therefore could not reach any definitive conclusions. Hematologic malignancies had the lowest survival rate. In comparison to Person L et al. (2021)’s study, our data showed lower survival rates, this may be due to broader grouping in our study. Their survival rate for carcinomas and lymphomas was over 85%, while ours was 72% for tumours of epithelial origin and 62% for tumours of hematologic malignancies. Rhabdomyosarcomas’ survival rate in Person L et al. (2021)’s study was 65–75% [10], while our data showed a slightly lower survival rate for muscular tumours at 63.6%.

When the hazard ratio was calculated, we found that muscle and hematologic-derived malignancies demonstrated an increased proportion, though it was not statistically significant. Curry SD et al. (2021) found, in a sub-distributional hazard analysis, that there was an improved disease-specific risk of death in the first year after diagnosis of rhabdomyosarcoma; however, their overall risk of death did not improve significantly [21]. Our hazard ratio analysis of tumour sites did not show a statistically significant difference compared with the reference category. We did not find papers to compare these findings to. We recommend performing further hazards ratio investigations in future studies.

Stage of cancer at the time of diagnosis was not evaluated in the current study because of missing data in over 60% of the cases. Evaluation of the data showed that there were many more cases that were registered since 2007. When possible, it is important for cancer registries to collect this data to enable future research in the field.

### Strengths and Limitations

Our study spans 47 years, which is a relatively long duration compared with other studies published in the field. Our study provided information regarding survival rates in many types of cancers, including both site and location. In addition, any epidemiologic study in this field is valuable, as it contributes to the global literature in an area where cases are relatively rare.

The limitations in our study mostly derive from the type of study: a retrospective epidemiological study that is based on a national registry.

As described previously, our study was based on the National Health registry from 1970 to 2017. Throughout the years there have been changes in ICD codes and in data completeness, etc. The registry updates the data according to current codes, but such changes can be accompanied with missing and/or unprecise data. Although this is indeed a limitation, it is true regarding all registries that are old enough to need updating.

Another limiting factor of this study was the number of individuals. Head and neck cancers amongst the paediatric population are rare, and therefore the sample size, especially when divided into subgroups, can lead to small cohorts that restrict the statistical power of the analysis and limit the ability to draw definitive conclusions.

In addition, the registry does not include data regarding treatment; this information could be beneficial in order to better understand which treatments allow higher survival rates.

Finally, as described earlier, missing data regarding stage of cancer did not allow analysis of cancer staging.

## 5. Conclusions

In this study we performed an analysis of data regarding head and neck cancers in patients aged 0–18 between the years 1970 and 2017, as collected from the Israel cancer registry.

We see research in this field as a crucial pursuit, as it helps provide information to clinicians and helps build stronger awareness.

Our analysis was divided by cancer histology and cancer site, and we searched for trends regarding gender, age, ethnicity, and origin. Later, we investigated the overall trend of cases diagnosed per year and found that there was no notable change in incidence. Finally, we calculated the survival rates of the cancers and found that the lowest survival rates were among cancers located on the oropharynx, or originating from a hematologic malignancies. We found that muscles and hematologic malignancies were associated with an increased risk of death compared with the reference group, although this finding was not statistically significant; further research in this field is recommended. We recommend conducting further research into the staging of cancers in the future and suggest that the registries make sure to collect this data when possible.

## Figures and Tables

**Table 1:** Cancer site distribution by gender, age, ethnicity and origin.

		Total		*p*	Oral Cavity		Oropharynx		Salivary Glands		Nasopharynx		Other	
		N	%		N	%	N	%	N	%	N	%	N	%
Gender	Male	236	60.10%	<0.001	26	6.60%	48	12.20%	37	9.40%	113	28.80%	12	3.10%
	Female	157	39.90%		29	7.40%	11	2.80%	47	12.00%	48	12.20%	22	5.60%
Age	<6	67	17.00%	<0.001	14	3.60%	16	4.10%	7	1.80%	24	6.10%	6	1.50%
	6–10	66	16.80%		9	2.30%	20	5.10%	12	3.10%	14	3.60%	11	2.80%
	10–13	45	11.50%		5	1.30%	9	2.30%	8	2.00%	22	5.60%	1	0.30%
	13–15	60	15.30%		12	3.10%	2	0.50%	14	3.60%	27	6.90%	5	1.30%
	15–17	74	18.80%		10	2.50%	6	1.50%	21	5.30%	33	8.40%	4	1.00%
	>17	81	20.60%		5	1.30%	6	1.50%	22	5.60%	41	10.40%	7	1.80%
Ethnicity	Jewish	314	79.90%	0.613	47	12.00%	47	12.00%	69	17.60%	123	31.30%	28	7.10%
	Other	79	20.10%		8	2.00%	12	3.10%	15	3.80%	38	9.70%	6	1.50%
Origin	E. Europe	1	0.30%	0.212	1	0.30%	0	0.00%	0	0.00%	0	0.00%	0	0.00%
	Israel	269	68.40%		44	11.20%	35	8.90%	53	13.50%	115	29.30%	22	5.60%
	Asia/Africa	5	1.30%		0	0.00%	0	0.00%	1	0.30%	3	0.80%	1	0.30%
	America/W. Europe/Australia	17	4.30%		0	0.00%	5	1.30%	5	1.30%	5	1.30%	2	0.50%
	Unknown	101	25.70%		10	2.50%	19	4.80%	25	6.40%	38	9.70%	9	2.30%
Total		393	100%		55	14.00%	59	15.00%	84	21.40%	161	41.00%	34	8.70%

Abbreviations are defined as follows: N = number, % = percentage from total, *p* = *p*-value, defined as <0.05, E. = eastern, W. = western.

**Table 2:** Distribution of cancer histology by gender, age, ethnicity and origin.

		Total		*p*	Epithelial Origin		Gland-Associated Tumours		Connective Tissue		Muscle		Nerve-Associated		Hematologic Malignancies		Other	
		N	%		N	%	N	%	N	%	N	%	N	%	N	%	N	%
Gender	male	236	60.10%	<0.001	94	23.90%	35	8.90%	4	1%	17	4.30%	2	0.50%	70	17.80%	14	3.60%
	female	157	39.90%		36	9.20%	50	12.70%	5	1.30%	16	4.10%	3	0.80%	28	7.10%	19	4.80%
Age	<6	67	17%	<0.001	6	1.50%	2	0.50%	3	0.80%	18	4.60%	1	0.30%	30	7.60%	7	1.80%
	6–10	66	16.80%		9	2.30%	13	3.30%	2	0.50%	12	3.10%	2	0.50%	22	5.60%	6	1.50%
	10–13	45	11.50%		19	4.80%	7	1.80%	0	0.00%	1	0.30%	0	0.00%	14	3.60%	4	1.00%
	13–15	60	15.30%		26	6.60%	19	4.80%	3	0.80%	1	0.30%	1	0.30%	7	1.80%	3	0.80%
	15–17	74	18.80%		27	6.90%	22	5.60%	1	0.30%	0	0.00%	1	0.30%	16	4.10%	7	1.80%
	>17	81	20.60%		43	10.90%	22	5.60%	0	0.00%	1	0.30%	0	0.00%	9	2.30%	6	1.50%
Ethnicity	Jewish	314	79.90%	0.231	99	25.20%	75	19.10%	8	2.00%	29	7.40%	4	1.00%	74	18.80%	25	6.40%
	Other	79	20.10%		31	7.90%	10	2.50%	1	0.30%	4	1.00%	1	0.30%	24	6.10%	8	2.00%
Origin	E. Europe	1	0.30%	0.82	1	0.30%	0	0.00%	0	0.00%	0	0.00%	0	0.00%	0	0.00%	0	0.00%
	Israel	269	68.40%		89	22.60%	51	13.00%	8	2.00%	24	6.10%	3	0.80%	65	16.50%	29	7.40%
	Asia/Africa	5	1.30%		2	0.50%	1	0.30%	0	0.00%	0	0.00%	1	0.30%	0	0.00%	1	0.30%
	America/W. Europe/Australia	17	4.30%		4	1.00%	5	1.30%	0	0.00%	3	0.80%	0	0.00%	4	1.00%	1	0.30%
	Unknown	101	25.70%		34	8.70%	28	7.10%	1	0.30%	6	1.50%	1	0.30%	29	7.40%	2	0.50%
Total		393	100.00%		130	33.10%	85	21.60%	9	2.30%	33	8.40%	5	1.30%	98	24.90%	33	8.40%

Abbreviations are defined as follows: N = number, % = percentage from total, *p* = *p*-value, defined as <0.05, E. = eastern, W. = western.

**Table 3:** Multivariable Cox proportional hazards model table.

Covariate	HR	95% CI (Lower)	95% CI (Upper)	*p*-Value
Diagnosis_Age	1.00	0.94	1.06	0.951
Female	1.05	0.60	1.82	0.865
Site—Oropharynx	0.36	0.12	1.10	0.074
Site—Salivary glands	0.56	0.15	2.16	0.403
Site—Nasopharynx	0.91	0.42	2.02	0.826
Site—Other	0.42	0.09	2.02	0.281
Histology—Epithelial	3.10	0.70	13.66	0.135
Histology—Gland-associated	0.48	0.06	3.79	0.483
Histology—Connective tissue	3.25	0.44	24.02	0.249
Histology—Muscle	5.56	1.10	28.04	0.038
Histology—Nerve-associated	0.00	0.00	∞	0.996
Histology—Hematologic malignancies	4.04	0.89	18.39	0.071

## Data Availability

The dataset presented in this article is not readily available because it contains confidential patient information. Requests to access the anonymous dataset should be directed to the corresponding author.
